# Association between job lost and mental health outcomes during the COVID-19 pandemic and the role of food insecurity as mediator of this relationship

**DOI:** 10.1590/0102-311XEN110523

**Published:** 2024-06-14

**Authors:** Fernanda de Oliveira Meller, Micaela Rabelo Quadra, Leonardo Pozza dos Santos, Samuel C. Dumith, Fernanda Daminelli Eugenio, Tamara Justin da Silva, João Vitor Santana Mendes, Antônio Augusto Schäfer

**Affiliations:** 1 Universidade do Extremo Sul Catarinense, Criciúma, Brasil.; 2 Universidade Federal de Pelotas, Pelotas, Brasil.; 3 Universidade Federal do Rio Grande, Rio Grande, Brasil.

**Keywords:** Work, Mental Health, Food Insecurity, COVID-19, Cross-Sectional Studies, Trabalho, Saúde Mental, Insegurança Alimentar, COVID-19, Estudos Transversais, Trabajo, Salud Mental, Inseguridad Alimentaria, COVID-19, Estudios Transversales

## Abstract

This study aimed to evaluate the association between employment status and mental health, considering food insecurity as a mediator of this relation. A cross-sectional population-based study was conducted with adults (≥ 18 and < 60 years) during the COVID-19 outbreak in two cities from Southern Brazil. Employment status was categorized into working, not working, and lost job. The mental health outcomes evaluated were depressive symptoms, perceived stress, and sadness. Food insecurity was identified by the short-form version of the *Brazilian Food Insecurity Scale*. Adjusted analyses using Poisson regression were performed to assess the association between employment status and mental health. Mediation analysis was performed to investigate the direct and indirect effects of employment status on mental health outcomes. In total, 1,492 adults were analyzed. The not working status was associated with 53% and 74% higher odds of perceived stress and of sadness, respectively. Being dismissed during the pandemic increased the odds of depressive symptoms, perceived stress, and sadness by 68%, 123%, and 128%, respectively. Mediation analyses showed that food insecurity was an important mediator of the association between employment status and depressive symptoms and sadness, but not of perceived stress. The complexity of these results highlights economic and nutritional aspects involved in mental health outcomes.

## Resumo

O objetivo foi avaliar a associação entre situação de trabalho e saúde mental e o papel da insegurança alimentar como mediadora dessa relação. Um estudo transversal de base populacional foi conduzido com adultos (≥ 18 e < 60 anos) durante o surto de COVID-19 em duas cidades do Sul do Brasil. A situação de trabalho foi categorizada em trabalhando, não trabalhando e perda do emprego. Os desfechos de saúde mental avaliados foram sintomas depressivos, percepção de estresse e sentimento de tristeza. A insegurança alimentar foi identificada pela versão reduzida da *Escala Brasileira de Insegurança Alimentar*. Análises ajustadas por regressão de Poisson foram realizadas para avaliar a associação entre situação de trabalho e saúde mental. A análise de mediação foi realizada para investigar os efeitos diretos e indiretos da situação de trabalho sobre os desfechos de saúde mental. No total, foram analisados 1.492 adultos. Não trabalhar associou-se a 53% mais chances de percepção de estresse e 74% maiores de tristeza. A perda do emprego aumentou as chances de sintomas depressivos, estresse percebido e sentimento de tristeza em 68%, 123% e 128%, respectivamente. As análises de mediação mostraram que a insegurança alimentar foi um importante mediador da associação entre situação de trabalho e sintomas depressivos e sentimento de tristeza, mas não para o estresse percebido. A complexidade desses resultados destaca aspectos econômicos e nutricionais envolvidos nos desfechos em saúde mental.

## Resumen

El objetivo fue evaluar la asociación entre la situación laboral y la salud mental y el papel de la inseguridad alimentaria como mediador en esta relación. Se realizó un estudio transversal basado en la población con adultos (≥ 18 y < 60 años) durante el brote de COVID-19 en dos ciudades del Sur de Brasil. La situación laboral se clasificó en trabajando, no trabajando y pérdida de empleo. Los resultados de salud mental evaluados fueron síntomas depresivos, percepción de estrés y sensación de tristeza. La inseguridad alimentaria fue identificada por la versión reducida de la *Escala Brasileña de Inseguridad Alimentaria*. Se realizaron análisis ajustados por regresión de Poisson para evaluar la asociación entre la situación laboral y la salud mental. Se realizó un análisis de mediación para investigar los efectos directos e indirectos de la situación laboral en los resultados de salud mental. En total, se analizaron 1.492 adultos. No trabajar se asoció con un 53% más de probabilidades de percepción de estrés y un 74% más de probabilidades de tristeza. La pérdida del trabajo aumentó las probabilidades de síntomas depresivos, estrés percibido y sentimientos de tristeza en un 68%, 123% y 128%, respectivamente. Los análisis de mediación mostraron que la inseguridad alimentaria era un mediador importante de la asociación entre la situación laboral y los síntomas depresivos y los sentimientos de tristeza, pero no para el estrés percibido. La complejidad de estos resultados destaca los aspectos económicos y nutricionales que intervienen en los resultados de salud mental.

## Introduction

The COVID-19 pandemic has become a public health problem due to numerous outcomes related to spreading dynamics, effects on public health, and prejudice towards mental health and to the population’s well-being, as well as its economic consequences ^1^ ^,^ ^2^ ^,^ ^3^. One of the economic consequences of the COVID-19 outbreak was the reduction in working hours, work absences, as well as discharges, affecting almost 2.7 billion workers worldwide ^2^.

Unemployment rate in Brazil, which had already been high since 2014 (7.2%) because of political-economic crisis, increased considerably during the pandemic ^4^ ^,^ ^5^. By late 2019 (before the pandemic) 11.1% of the population was unemployed, which increased to 14.9% in 2020 (July to September) ^5^.

Since 2013, the prevalence of food insecurity increased significantly in the Brazilian territory, along with the political-economic crisis and unemployment rate. The most significant increase occurred in mild food insecurity, in which an increase in prevalence of 9.2 percentage points was observed from 2013 (14.8%) to 2018 (24%). In the same period, moderate food insecurity prevalence increased from 4.6% to 8.1%, and severe food insecurity from 3.2% to 4.6% ^6^. In 2020, food insecurity in Brazil reached around 116.8 million people, with prevalence of mild, moderate, and severe levels reaching 34.7%, 11.5%, and 9%, respectively ^7^.

Both employment status and food insecurity have been identified as determinants of mental health outcomes. During the COVID-19 pandemic, unemployment was associated with suicide ^8^, perceived stress ^3^, anxiety, concern, loss of interest, and depression ^9^. Furthermore, living in Brazil was associated with a higher risk of depressive symptoms and anxiety, when compared to living in Spain, in a study conducted with essential workers (health, transportation, food or cleaning workers), suggesting that living in a middle-income country, with large social inequalities, can harm mental health ^10^.

A household member losing their job, reduction in household income, increased family indebtedness, and the need to cut essential expenses are conditions that affected food access in Brazil. In the case of a family member losing their job, the prevalence of severe food insecurity during COVID-19 pandemic reached 19.8% ^11^. As an aggravating factor, it is known that food insecurity during the pandemic has been associated with mental health issues, such as stress, anxiety, and depressive symptoms ^12^ ^,^ ^13^ ^,^ ^14^.

Therefore, considering the multiple impacts of the COVID-19 pandemic on people’s well-being, affecting economic factors and mental health, we aimed to evaluate the association between employment status and mental health outcomes, using data from population-based studies conducted with adults from Southern Brazil during the COVID-19 pandemic. We further investigated whether such association was mediated by food insecurity.

## Methods

### Study design and sample

This cross-sectional research used data from the *Mental Covid: Impact of COVID-19 on the Mental Health of the Population* study, conducted during the COVID-19 outbreak, from October 2020 to January 2021. The study was carried out in two cities from Southern Brazil: Criciúma (Santa Catarina State) and Rio Grande (Rio Grande do Sul State). Criciúma has an estimated population of 217,311 inhabitants, with a Human Development Index (HDI) of 0.788, and population density around 815.87 inhabitants per km^2^ ^15^. Rio Grande has an estimated population of 211,965 inhabitants, with a HDI of 0.744, and population density of 72.79 inhabitants per km^2^ ^15^.

Sampling process was conducted in two stages, based on data from the *2010 Brazilian Demographic Census* ^16^: the primary units were composed of the census sector, and the secondary units were the households. Firstly, the census sectors were randomly selected. Then, the households were randomly chosen according to the previously selected census sectors. In Criciúma, 60 census tracts were sampled, from a total of 307, resulting in 15,765 households, of which 607 were included in this study. In Rio Grande, 90 census tracts were sampled, resulting in 29,734 households, of which 900 were systematically selected. All adults aged 18 years old or more living in the selected households were invited to participate, totaling 2,170 individuals (1,307 adults in Rio Grande and 863 adults in Criciúma). Subjects with a physical and/or mental impairment and those who were unable to complete the survey were excluded. Considering that employment status was the main exposure of this study, only economically active individuals aged 18-64 were included, as defined by the Brazilian Institute of Geography and Statistics (IBGE, acronym in Portuguese). Individuals aged 65 years or older were excluded from the analysis.

### Data collection

Data collection was performed using a single, pre-coded, standardized questionnaire, including information about mental health disorders, behavior during the pandemic, quality of life, nutrition, physical activity, and chronic diseases. The questionnaire was applied by face-to-face interviews with trained personnel. All interviewers wore personal protective equipment during fieldwork to avoid SARS-CoV-2 infection. Questionnaire application lasted 30 minutes, on average, and was performed using tablet computers and REDCap application (https://redcapbrasil.com.br/).

### Studied variables

### Employment status

To evaluate employment status during the COVID-19 pandemic the following question was used: “How has COVID-19 social distancing affected your occupation/work?”, with the answer options: (1) I did not work before the pandemic and I have continued not working during the pandemic, (2) I have continued working, (3) I have continued working but at home (remote work), (4) I have started working during the pandemic, (5) I lost my job during the pandemic. Then, employment status was divided into three categories for analyses purposes. Individuals who chose option 1 were classified as “not working”. Individuals who chose options 2, 3, or 4 were grouped in “working” category, and those who chose the last option (option 5) were classified as “lost job”.

### Mental health outcomes

The mental health problems evaluated were depressive symptoms, perceived stress, and feelings of sadness. Depressive symptoms were assessed by the *Patient Health Questionnaire-9* (PHQ-9), which has been validated for Brazilian population. This tool investigates the frequency of depressive symptoms (depressive mood, anhedonia, trouble sleeping, tiredness or lack of energy, change in appetite or weight, feeling of guilt or uselessness, trouble concentrating, feeling slow or agitated, and thoughts about death or suicidal ideation) in the two weeks prior to the interview. Each of the 10 questions of the questionnaire is scored from 0 to 3, corresponding to “never”, “less than once per week”, “once per week or more”, and “almost every day” ^17^.

The cut-off point of ≥ 9 proposed by Santos et al. ^17^ was adopted due to its high sensitivity and specificity, and previous application in other population-based studies in Brazil ^18^ ^,^ ^19^. Individuals who scored 9 or more in the test were then considered with positive screening for depressive symptoms.

Perceived stress was assessed using the *Perceived Stress Scale* (PSS-14), previously validated for the Brazilian population ^20^. The PSS-14 is a 14-item scale that assesses stressful experiences in the month prior to the interview. This scale creates a score ranging from 0 to 56 points. To define perceived stress in this study, the score was categorized into quintiles, in which individuals classified in the fourth or fifth quintiles were considered as stressed. Perceived stress was already defined by this approach in previous studies ^21^ ^,^ ^22^.

Finally, feelings of sadness were assessed using the faces scale proposed by Andrews & Withey ^23^. This scale includes seven face options, allowing interviewees to classify their feeling from very happy to very sad. Individuals who chose faces 5, 6, or 7 were considered as having feelings of sadness ^23^.

### Household food insecurity status

The short-form version of the *Brazilian Food Insecurity Scale* (EBIA, acronym in Portuguese) was used to evaluate food insecurity. This scale is composed of five questions, considering a 3-month recall period. The scale includes aspects of concern about not having enough food to eat, food availability as well as impaired diet quality. While the scale does not categorize the severity of food insecurity (mild, moderate, and severe), it helps to screen households experiencing it, since the scale presented high sensitivity and specificity when compared to the complete version of the EBIA ^24^. The short-form version was chosen over the complete questionnaire to minimize required time for questionnaire administration in each household, considering the pandemic scenario. Households in which individuals gave at least one positive answer were classified as experiencing food insecurity.

### Covariables

Sociodemographic variables were included as potential confounders of the relationship between job situation and mental health outcomes. The variables included were sex (male, female), age (collected in completed years and categorized as 18-39, 40-59, 60 or more), marital status (married, single, divorced/widowed), schooling level (primary education, secondary education, tertiary education), and wealth index (by the analysis of main components, with the variables: number of rooms, bathrooms, freezer, clothes dryer, computer, air conditioning, cars, and internet access in the household; subsequently categorized in tertiles).

### Statistical analysis

Descriptive analyses of the sociodemographic variables were performed, presenting absolute (n) and relative (%) frequencies and their respective 95% confidence intervals (95%CI). Crude analysis of the association between mental health outcomes and employment status was performed using the Fisher’s exact test, with a 5% significance level.

Adjusted analyses were also performed to assess whether the associations between employment status and mental health outcomes were independent of sociodemographic characteristics. Logistic regression was used, and the results were reported as odds ratio (OR) and its corresponding 95%CI. Variables with a 20% significance level (p-value < 0.20) were considered confounders and remained in the final model.

The possible role of food insecurity as mediator of the association between employment status and mental health outcomes was evaluated. The method proposed by Erikson et al. ^25^ was used to investigate the direct and indirect effects of employment status on mental health, for this method allows for decomposing the total effect of a categorical variable with binomial distribution into direct and indirect effects ^26^. So, the overall effect of employment status on metal health outcomes was decomposed into direct and indirect effects, considering food insecurity as the mediator. Also, the magnitude and the statistical significance level of the indirect effect were estimated, as well as its proportion of the total association. The presence of a significant indirect effect suggested that food insecurity plays a mediating role in the investigated relationship. Standard errors were estimated using the bootstrap test with 1,000 replications ^27^. Mediation analyses were adjusted for the same confounders included in logistic regression models. The results were shown as OR from the extraction of the obtained exponential coefficient.

The statistics program Stata version 16.1 (https://www.stata.com) was used to perform all analyses using *svy* prefix, which considers the complexity of the sampling process and the effect of study design.

### Ethical considerations

This research was previously evaluated and authorized by the Research Ethics Committee of the Federal University of Rio Grande in July 2020 (protocol n. 4.162.424; CAAE: 30955120.0.0000.5324). All study participants provided informed verbal consent.

## Results

From 2,170 interviewees in both cities, 1,681 economically active adults (18-64 years old) were included in the analysis. Most participants were female (59.3%; 95%CI: 56.9; 61.6) and around 10% were older than 60 years (95%CI: 9.8; 12.8). Approximately 50% were married (95%CI: 45.9; 50.7), and around 65% had complete secondary education. Finally, 35% of the sample were classified in the highest socioeconomic strata (95%CI: 33.2; 37.9) (Table 1).

**Table 1:** Employment status according to participants’ socioeconomic and demographic characteristics. Criciúma (Santa Catarina State) and Rio Grande (Rio Grande do Sul State), Brazil, 2021 (n = 1,681).

Characteristics	Total		Working	Not working	Lost job	p-value
	n	% (95%CI)	% (95%CI)	% (95%CI)	% (95%CI)	
Sex						< 0.001
Male	684	40.7 (38.4; 43.1)	70.0 (66.4; 73.4)	21.5 (18.5; 24.8)	8.5 (6.6; 10.9)	
Female	997	59.3 (56.9; 61.6)	49.4 (46.3; 52.6)	40.7 (37.7; 43.9)	9.8 (8.1; 11.9)	
Age (years)						< 0.001
18-39	729	43.4 (41.0; 45.8)	64.9 (61.3; 68.3)	25.7 (22.6; 29.0)	9.4 (7.5; 11.8)	
40-59	763	45.4 (43.0; 47.8)	59.3 (55.7; 62.9)	31.3 (28.0; 34.8)	9.3 (7.4; 11.7)	
60 or more	189	11.2 (9.8; 12.8)	23.6 (17.9; 30.4)	68.0 (60.8; 74.4)	8.4 (5.1; 13.5)	
Marital status						< 0.001
Married	812	48.3 (45.9; 50.7)	61.9 (58.5; 65.3)	30.3 (27.1; 33.6)	7.8 (6.1; 9.9)	
Single	697	41.5 (39.1; 43.8)	57.7 (53.9; 61.4)	31.3 (27.9; 35.0)	11.0 (8.8; 13.6)	
Divorced/Widowed	172	10.2 (8.9; 11.8)	39.3 (32.2; 46.9)	51.2 (43.7; 58.7)	9.5 (5.9; 15.0)	
Schooling level						< 0.001
Primary education	541	32.4 (30.2; 34.7)	43.5 (39.3; 47.8)	43.9 (39.7; 48.2)	12.6 (10.0; 15.7)	
Secondary education	619	37.1 (34.8; 39.5)	61.8 (57.9; 65.6)	29.3 (25.8; 33.1)	8.8 (6.8; 11.4)	
Tertiary education	508	30.5 (28.3; 32.7)	69.5 (65.2; 73.4)	24.3 (20.7; 28.4)	6.2 (4.4; 8.7)	
Wealth index (tertiles)						< 0.001
1st (lower living standard)	477	29.6 (27.5; 31.9)	49.9 (45.4; 54.4)	37.0 (32.8; 41.5)	13.0 (10.3; 16.4)	
2nd	561	34.9 (32.6; 37.2)	58.5 (54.3; 62.6)	32.4 (28.6; 36.5)	9.1 (7.0; 11.9)	
3rd (higher living standard)	571	35.5 (33.2; 37.9)	64.7 (60.6; 68.7)	29.3 (25.6; 33.3)	5.9 (4.2; 8.3)	

95%CI: 95% confidence interval.

Most individuals (57.8%) reported working during the COVID-19 pandemic, while 32.9% of the sample were not working, and 9.3% had been dismissed. Those who were not working by the time of interview were most likely women, older adults (60 years of age or more), widowed or divorced, with low formal education (primary education) and with lower wealth index. Regarding termination of employment, single individuals, those with primary education and with lower wealth index were more likely to lose their jobs amid COVID-19 pandemic (Table 1).

Perceived stress was the most common mental health variable observed in the sample, affecting 40.6% of participants; followed by feelings of sadness and depressive symptoms, with prevalence rates of 16.5% and 14.6%, respectively. Depressive symptoms and perceived stress were more prevalent among women, adults aged under 60, unmarried individuals and with lower wealth index. Additionally, women and individuals with lower schooling levels and lower wealth index were more likely to report feelings of sadness (Table 2).

**Table 2:** Mental health outcomes according to socioeconomic and demographic characteristics. Criciúma (Santa Catarina State) and Rio Grande (Rio Grande do Sul State), Brazil, 2021 (n = 1,681).

Characteristics	Depressive symptoms	Perceived stress	Feelings of sadness
% (95%CI)	% (95%CI)	% (95%CI)
Sex	p < 0.001	p < 0.001	p < 0.001
Male	7.6 (5.8; 9.8)	33.1 (29.6; 36.7)	10.4 (8.3; 12.9)
Female	19.4 (17.1; 22.0)	45.8 (42.7; 48.9)	20.8 (18.4; 23.4)
Age (years)	p = 0.043	p = 0.003	p = 0.110
18-39	14.6 (12.2; 17.3)	44.4 (40.8; 48.0)	15.1 (12.7; 17.9)
40-59	16.0 (13.6; 18.8)	39.3 (35.9; 42.9)	18.6 (16.0; 21.5)
60 or more	9.0 (5.7; 14.0)	31.2 (25.0; 38.2)	13.8 (9.5; 19.4)
Marital status	p = 0.011	p = 0.005	p = 0.976
Married	12.0 (9.9; 14.4)	37.2 (33.9; 40.5)	16.4 (14.0; 19.1)
Single	17.3 (14.6; 20.3)	45.3 (41.6; 49.0)	16.8 (14.2; 19.7)
Divorced/Widowed	16.3 (11.5; 22.6)	38.0 (31.0; 45.5)	16.3 (11.5; 22.6)
Schooling level	p = 0.248	p = 0.885	p < 0.001
Primary education	14.1 (11.4; 17.3)	40.8 (36.7; 45.0)	21.6 (18.4; 25.3)
Secondary education	13.3 (10.8; 16.2)	39.7 (58.8; 43.6)	11.5 (9.2; 14.2)
Tertiary education	16.7 (13.7; 20.2)	41.0 (36.8; 45.4)	16.9 (13.9; 20.4)
Wealth index (tertiles)	p = 0.353	p < 0.001	p = 0.219
1st (lower living standard)	16.1 (13.1; 19.7)	52.6 (48.1; 57.1)	17.8 (14.6; 21.5)
2nd	14.5 (11.8; 17.6)	38.7 (34.8; 42.8)	16.6 (13.7; 19.9)
3rd (higher living standard)	13.0 (10.5; 16.0)	33.4 (29.6; 37.4)	14.0 (11.4; 17.1)

95%CI: 95% confidence interval.

**Figure 1:**
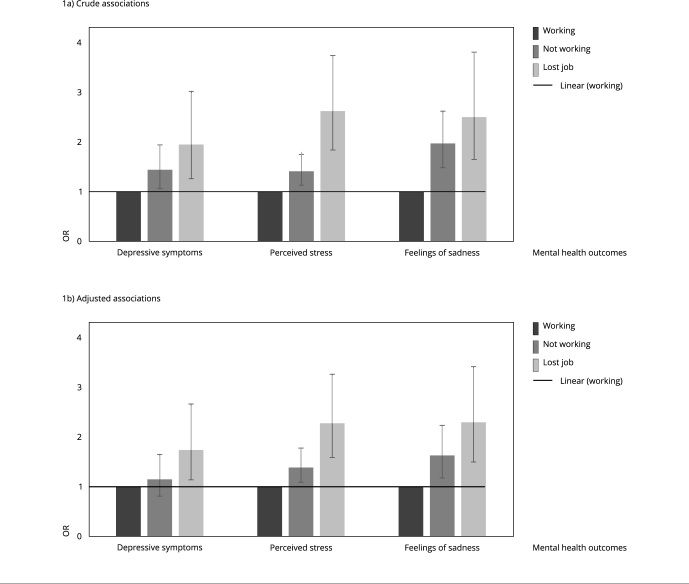
Crude and adjusted associations between employment status and mental health outcomes during the COVID-19 pandemic. Criciúma (Santa Catarina State) and Rio Grande (Rio Grande do Sul State), Brazil, 2021.

Figure 1 shows crude and adjusted associations between employment status and mental health outcomes. The odds of presenting depressive symptoms, perceived stress and sadness were higher in individuals who were not working or who lost their job during the pandemic (Figure 1a). Adjustment for confounders did not change the associations. Not working before and during the COVID-19 pandemic was associated with higher odds of perceived stress (OR = 1.39; 95%CI: 1.10; 1.78) and feelings of sadness (OR = 1.63; 95%CI: 1.18; 2.25), while termination of employment during the pandemic increased the odds of depressive symptoms (OR = 1.74; 95%CI: 1.14; 2.67), perceived stress (OR = 2.28; 95%CI: 1.59; 3.27), and feelings of sadness (OR = 2.30; 95%CI: 1.50; 3.52), regardless of the potential confounders included in the analysis (Figure 1b).

OR: odds ratio.

About one third of the sample reported living in food insecurity situation amid COVID-19 pandemic (32%). Mediation analyses showed that food insecurity mediated the association of employment status with mental health outcomes. The effect of termination of employment on depressive symptoms mediated by food insecurity (indirect effect) represented 15.9% of the total effect. Regarding, the effect of termination of employment on perceived stress mediated by food insecurity, represented 7.8% of the total effect. Finally, the effect of employment status on sadness mediated by food insecurity represented almost 20% of the total effect. Also, food insecurity seemed to not mediate the association between not working and mental health outcomes (Table 3).

**Table 3:** Association between employment status and mental health outcomes during the COVID-19 pandemic mediated by food insecurity. Criciúma (Santa Catarina State) and Rio Grande (Rio Grande do Sul State), Brazil, 2021 (n = 1,681).

	Total effect *			Direct effect *		Indirect effect *		Mediation (%)
	OR (95%CI)	p-value	OR (95%CI)	p-value	OR (95%CI)	p-value	
Outcome: depressive symptoms							
Working	1.00		1.00		1.00		
Not working	1.27 (0.9; 1.78)	0.169	1.22 (0.87; 1.71)	0.241	1.04 (1.00; 1.08)	0.066	15.6
Lost job	1.69 (1.06; 2.69)	0.026	1.54 (0.96; 2.45)	0.071	1.10 (1.01; 1.20)	0.036	18.3
Outcome: perceived stress							
Working	1.00		1.00		1.00		
Not working	1.48 (1.15; 1.90)	0.002	1.45 (1.13; 1.87)	0.004	1.02 (0.99; 1.05)	0.141	5.0
Lost job	2.24 (1.53; 3.29)	< 0.001	2.13 (1.45; 3.12)	< 0.001	1.05 (0.99; 1.12)	0.096	6.6
Outcome: feelings of sadness							
Working			1.00		1.00		
Not working	1.80 (1.31; 2.47)	< 0.001	1.70 (1.24; 2.33)	0.001	1.06 (1.01; 1.11)	0.019	9.7
Lost job	2.37 (1.52; 3.69)	< 0.001	2.04 (1.30; 3.19)	0.002	1.16 (1.06; 1.27)	0.001	17.3

95%CI: 95% confidence interval; OR: odds ratio.

* Adjusted for sex, age, marital status, schooling level, and wealth index.

## Discussion

This study investigates the association between employment status and mental health outcomes during the COVID-19 pandemic in adults, assessing whether this relation is mediated by food insecurity. Results showed that individuals who were not working presented higher odds of perceived stress and feelings of sadness. Also, depressive symptoms, perceived stress, and feelings of sadness are more likely to occur among those who lost their job during the COVID-19 pandemic.

In addition, food insecurity seems to mediate the association between termination of employment and mental health outcome in the economically active sample of this study. The positive association between termination of employment and mental health outcomes came from both direct and indirect effects, suggesting that food insecurity may be an important pathway linking termination of employment to mental health outcomes.

Mental health can be profoundly influenced in this scenario. Previous studies have shown that termination of employment was related to higher frequency of perceived stress ^3^, anxiety, concern, loss of interest, and depression ^9^. The cross-sectional study by McDowell et al. ^28^ with American adults showed that termination of employment in that population was associated with higher prevalence of depression, anxiety, and stress, mainly in individuals who became unemployed due to the COVID-19 pandemic. Similarly, in Australia, baseline analysis from a cohort study with adults found that negative changes in work situation, such as reduction in working hours, leaves of absence, and termination of employment, were related to higher psychological distress and poor mental health ^29^.

In the United States, a cross-sectional study with individuals ≥ 18 years of age demonstrated that those who lost their jobs experienced more frequent mentally unhealthy days. Even individuals who were under leave of absence, which is associated with anxiety increase, showed better mental health than those who lost their jobs ^29^. This is an important aspect of the relationship between employment status and mental health, since the effect measure of the associations found in our study was always higher when the exposure variable was termination of employment, indicating that this variable may impact mental health more than, for example, not working.

Therefore, it is important to define and differ termination of employment and the status of not working or unemployment. While termination of employment is easily defined as involuntary dismiss of one’s job by the employing company, unemployment is defined as lack of a job, whose previous discharge has been voluntary or forced, and in which the individual is currently seeking a new one ^30^. Both are accompanied by significant changes and prejudices in life and health of the affected ones. However, the stronger effects of these changes, especially those beyond economic aspects, are observed in termination of employment ^30^. This might explain the higher frequency of the associations in individuals who lost their jobs than among those who were not working.

The effect of different forms of unemployment on negative mental health outcomes has already been explored in the literature, with results confirming such association ^31^. The meta-analysis developed by Paul & Moser ^31^ also highlights that negative effects are more significant in scenarios with economic difficulties, such as in underdeveloped countries and with unequal income distribution.

Studies from Australia and the United States indicate that perceived financial stress and concern (issues related to family and household maintenance) mediate the association between termination of employment and negative mental health outcomes, during the COVID-19 pandemic ^32^ ^,^ ^33^. Unemployment rate is associated with food insecurity among the population during economic recession, since loss of income has a great influence in food access and quality ^34^ ^,^ ^35^ ^,^ ^36^. At the same time, studies conducted during the pandemic demonstrated that food insecurity and poor mental health are related ^13^ ^,^ ^36^ ^,^ ^37^. Thus, we hypothesized that food insecurity may play an important role in the association between employment status and mental health.

To sustain this hypothesis, we conducted further analyses, aiming to assess whether this relationship is mediated by household food insecurity. Results showed that food insecurity mediated only the association between termination of employment and depressive symptoms, in which the effect of the former on the latter was associated with food insecurity. Additionally, food insecurity also mediated employment status and sadness, but in this case the effect came from both direct and indirect pathways.

The literature also indicates the relationships between food insecurity and financial distress, and between food insecurity and negative mental health outcomes. During financial crises in Brazil, food insecurity intensely affected the poorest individuals, increasing the occurrence of such outcomes ^38^. On the other hand, food insecurity is also related to impairment of mental well-being, with its presence associated with an increase in anxiety, depression, and stress ^22^ ^,^ ^39^ ^,^ ^40^. Fang et al. ^39^ study raised the hypothesis of food insecurity affecting mental health more than termination of employment during the COVID-19 pandemic, which emphasizes food insecurity as a mediator of employment status and mental health.

Such mediation may refer to the intertwined pathways between food insecurity and financial status. Food insecurity can be defined as the impossibility of accessing food in proper quantity and quality due to lack of resources. Therefore, financial resources are important to food security maintenance, and when nonexistent or reduced could lead to eating difficulties and, consequently, cause mental health damage ^41^.

Another possible explanation for this mediation is diet quality, for its involvement in depression pathophysiology. Inflammation and oxidative stress, as well as hypothalamic-pituitary-adrenal axis dysfunction, tryptophan metabolism, central nervous system and gut microbiota effects can be developed and cause depression based on the amount of vitamins, minerals, and other bioactive compounds in the diet ^37^. Socioeconomic status is a determinant factor to one’s diet quality. Therefore, it is possible to establish that diet quality is affected by work and food security status ^42^. Thus, individuals with lower socioeconomic status might have a diet that favors depressive symptoms, since it is commonly composed of low nutritional values ^41^ ^,^ ^42^.

This study holds some methodological limitations. It is not possible to establish causality of the associations due to the cross-sectional design. Therefore, there may have been a reverse causality bias in the results, for it is also possible that individuals with poor mental health before and during the COVID-19 pandemic were less likely to be able to work. In other words, it hampers to define whether employment status influences the occurrence of mental health disorders, or whether previous mental health conditions influence the maintenance of a job. Similar phenomenon has already been mentioned in a previous study ^43^. Moreover, mental health variables were evaluated through screening tools and not using diagnostic methods, for it is unfeasible to apply diagnostic tools in population-based studies, especially in a public health emergency scenario. Thus, these tools are important instruments for epidemiological studies and have been previously used ^3^ ^,^ ^9^ ^,^ ^17^ ^,^ ^20^ ^,^ ^23^ ^,^ ^44^.

Positive points to the study are population-based data collection conducted in two cities from Southern Brazil with a representative sample of urban population. Furthermore, the interviews were conducted face-to-face at the participants’ households. This is a remarkable strength of this investigation, since most studies carried out during the COVID-19 pandemic collected data online.

In conclusion, employment status seems to be associated with mental health, mediated by food insecurity. Individuals who were not working during the COVID-19 pandemic presented higher odds of perceived stress and sadness, while those who lost their job presented higher prevalence of depressive symptoms, perceived stress, and sadness. Considering the complex outcomes of this study, which relates economic and nutritional aspects to mental health, future longitudinal prospective studies are needed to better understand the causal pathways on the relationship between employment status, food insecurity, and mental health; such studies are even more relevant if with add the concerning scenario of growth in mental health conditions throughout Brazil.
